# The Effect of Tissue Oxygen Tension on the Radiosensitivity of Leukaemia Cells Irradiated In Situ in the Livers of Leukaemic Mice

**DOI:** 10.1038/bjc.1959.75

**Published:** 1959-12

**Authors:** H. B. Hewitt, C. W. Wilson


### THE EFFECT OF TISSUE OXYGEN TENSION ON THE RADIO-SENSITIVITY OF LEUKAEMIA CELLS IRRADIATED *IN SITU* IN THE LIVERS OF LEUKAEMIC MICE

H. B. HEWITT AND C. W. WILSON

*From the Westminster Hospital Medical School, London, S.W.1*

Received for publication August 29, 1959

A DETAILED description of the lymphocytic type of leukaemia of CBA mice used in the present study, including an assay method for determining the density of viable leukaemia cells in single-cell suspensions prepared from the infiltrated livers of fully leukaemic mice, was given in a previous paper (Hewitt, 1958). The assay method was later used to determine a survival curve for the liver leukaemia cells irradiated *in vivo* in leukaemic mice breathing air during total-body irradiation (Hewitt and Wilson, 1959). Under these conditions a linear relationship was demonstrated between whole-body radiation dose and log survival ratio among the viable leukaemia cells. The $D_0$ value given by the linear part of the curve (the increment in dose of radiation required to reduce the number of viable leukaemia cells to 37 per cent) was 165 r $^{60}$Co gamma radiation. The disposition of the points suggested a 2-hit curve but this feature was not determined with certainty. Comparison of the $D_0$ value for mouse leukaemia cells with the $D_0$ value obtained for human HeLa cells irradiated *in vitro* under well oxygenated conditions (Puck and Marcus, 1956) could not be usefully made without information concerning the oxygen tension in the environment of the mouse leukaemia cells at the time of their irradiation *in vivo* in mice breathing air. Comparison of the radiosensitivities of the HeLa cells and mouse leukaemia cells, as described by the established $D_0$ values, would only be valid if it could be shown that the leukaemia cells were, like the HeLa cells, in a well-oxygenated environment at the time of their irradiation. The importance of this comparison relates to the appropriateness of extrapolation to human tumours of radiobiological data obtained for mouse tumours.

The macroscopic and histological appearances of the livers of the fully leukaemic mice used for determination of the *in vivo* survival curve already referred to suggested that the vascularity of the liver is impaired at this stage of the disease ; and it was considered that an unknown proportion of the masses of vigorously metabolising leukaemia cells infiltrating the liver might have been under severely hypoxic, if not anoxic, conditions at the time of irradiation. If this proportion were large, a rise in the radiosensitivity of the cells might be expected if the mice were exposed to radiation while breathing oxygen instead of air ; on the other hand, rendering the cells anoxic during irradiation, by killing the mouse before exposure, would not be expected to reduce the radiosensitivity significantly. Investigations into these questions form the subject of the present paper.

### MATERIALS AND METHODS

*Mice.*—CBA male mice bred in this laboratory by brother-to-sister mating were used in all the experiments ; the mice were 2–4 months old at the time of experiment. The leukaemic mice which were irradiated had been injected intraperitoneally with several million leukaemia cells 9–11 days previously. At the time of irradiation the mice were moderately sick, almost all organs being heavily infiltrated with leukaemia cells.

*Irradiation of mice.*—The leukaemic mouse was placed in a " Perspex " cylinder of such dimensions as permitted the mouse to assume a normal uncramped resting posture but prevented it from turning round. The cylinder was closed at each end with a rubber bung perforated by a short length of glass tubing, one end of which served as an exit for the gas mixture to be circulated through the cylinder. The gas mixture was allowed to flow in at the opposite end via a flow meter from a cylinder containing the desired gas mixture (British Oxygen Company, Ltd.). Two gas mixtures were used : air containing 5 per cent carbon dioxide, and oxygen containing 5 per cent carbon dioxide. The carbon dioxide was included to ensure an adequate respiratory stimulus. Each gas mixture was allowed to flow through the cylinder at a rate of 1·8–2·2 litres/min. for 10 minutes before, and throughout, irradiation. The " Perspex " cylinder containing the mouse to be irradiated was positioned in a beam of $^{60}$Co gamma radiation from a Kilocurie beam unit (a " Theratron "). The whole-body dose was delivered at a mean dose rate of 70–74 r/min. and was given as equal exposures to both sides of the cylinder. The distance from the source to the centre of the cylinder was 62 cm. and the field size used was such as to cover the mouse very generously. Under these conditions, the whole-body dose was uniform throughout the mouse to about $\pm 3$ per cent.

At each radiation dose level used, an air-breathing and an oxygen-breathing mouse, both at the same advanced stage of the disease, were exposed separately but on the same day, the two mice being treated under identical conditions except for the different gas mixtures respired. Experiments at different dose levels were done on different days, but mice at a similar stage of the disease were used on all occasions.

For irradiation of the leukaemia cells under what are assumed to be anoxic conditions leukaemic mice were killed by fracture of the neck 1 minute before the start of their exposure to radiation under the same conditions as the living mice. The series of dead mouse experiments was undertaken at a slightly later stage of the leukaemia's history than the living mouse experiments. However, the radiosensitivity of the cells in living mice was determined again after completion of the dead mouse experiments, and was found to be unchanged.

*Measurement of the survival ratios in irradiated leukaemia cell populations.*—Details of the method of preparing single-cell suspensions of leukaemia cells from the livers of leukaemic mice have been described previously (Hewitt, 1958). In the present experiments, such suspensions were prepared from the livers of the irradiated mice within 20 minutes of the end of their exposure. The density of morphologically intact, and *apparently* viable, leukaemia cells was determined by counting in a haemocytometer by phase-contrast microscopy. 0·2 ml. volumes of serial tenfold dilutions of the counted suspension in 5 per cent CBA mouse serum in Tyrode solution were injected intraperitoneally into groups of

6 CBA male mice. The range of mean cell doses injected was preselected to cover the expected end-point of an assay. The injected mice were observed for a period of 90 days (a period twice as long as the longest latent period ever observed in a mouse injected with a small inoculum of cells of this strain of leukaemia), and the incidence of leukaemic deaths was recorded for each group. From the results, the number of morphologically intact leukaemia cells required to transfer leukaemia to half a group of injected mice was calculated by the method of Reed and Muench (1938). It was found that the yield of morphologically intact leukaemia cells obtained from the livers of irradiated mice within one hour of irradiation was not reduced below that expected from untreated leukaemic mice. The TD50 values obtained for morphologically intact cells from irradiated mice, however were significantly higher than the average value given by cells from untreated mice and were a function of the dose of radiation. Thus, irradiation abolished the reproductive integrity of a proportion of the leukaemia cells without producing immediate morphological changes appreciable by phase-contrast microscopy. It was found previously (Hewitt, 1958) that in 6 assays of leukaemia cells from unirradiated leukaemic mice, the TD50 values varied from 0·7 to 3·0 cells, averaging 2·0 cells. The log survival ratio in an irradiated leukaemia cell population was calculated simply by subtracting the log TD50 given by the irradiated cell population from the log of the average TD50 given by unirradiated populations.

### RESULTS

### *Irradiation of leukaemia cells in mice breathing* 95 *per cent air or* 95 *per cent oxygen*

The log survival ratio among the liver leukaemia cells was determined for mice breathing oxygen containing 5 per cent carbon dioxide during irradiation with 800, 1400 or 2000 r total-body radiation. At each dose level the log survival ratio was similarly determined for the leukaemia cells irradiated in a mouse breathing air containing 5 per cent carbon dioxide. The results are recorded in Table I. In Fig. 1, the result of each experiment has been entered in the graph relating log survival ratio and radiation dose. The points obtained are seen in relation to the log survival curve previously obtained for the leukaemia cells irradiated in mice breathing air alone (Hewitt and Wilson, 1959). None of the points departs significantly from the log survival curve previously obtained, and there is no significant difference at each dose level between the survival ratios obtained for cells irradiated in mice breathing 95 per cent air and for cells irradiated in mice breathing 95 per cent oxygen.

TABLE I.—*Log Survival Ratios among Leukaemia Cells Irradiated* In Vivo *in the Livers of Leukaemic Mice Breathing* (a) 95 *per cent Oxygen,* (b) 95 *per cent Air, during Irradiation*

| Dose of radiation (r ; $^{60}$Co gamma rays) | Log survival ratio: Mice breathing oxygen | Log survival ratio: Mice breathing air |
|---|---|---|
| 800 | $\bar{3}\cdot 83$ | $\bar{3}\cdot 72$ |
| 1400 | $\bar{4}\cdot 55$ | $\bar{4}\cdot 90$ |
| 2000 | $\bar{5}\cdot 20$ | $\bar{6}\cdot 53$ |

*Irradiation of leukaemia cells in mice immediately after death*

It will be appreciated that in the dead mouse experiments the leukaemia cells are allowed to remain in the livers of the dead mice for a period slightly longer than the length of time occupied by the exposure to radiation, and that during this time they would be expected to be under strictly anaerobic conditions and at a temperature falling gradually from 37° C. to room temperature. It was conceivable that a proportion of the cells might lose their viability under these

FIG. 1.—Survival curves for leukaemia cells irradiated (*a*) in dead mice, (*b*) in living mice.
– – – – – Survival curve for cells irradiated in living mice breathing air (Hewitt and Wilson, 1959).
○ Survival ratios for cells irradiated in mice breathing 5 per cent $CO_2$ in oxygen.
△ Survival ratios for cells irradiated in mice breathing 5 per cent $CO_2$ in air.

conditions. If this were so, the viability loss for cells irradiated in dead mice would be the summation of loss due to anoxia and starvation, and loss due to radiation-induced damage. The longest time of irradiation in these experiments was under 43 minutes (when 3000 r was delivered at a rate of 70·6 r/min.). A preliminary experiment was therefore done in an attempt to detect a rise in the TD50 value given by the leukaemia cells after their residence in the liver of a dead leukaemic mouse at room temperature for 47 minutes after death. A portion of liver was removed from a leukaemic mouse immediately after death by neck fracture and the operation wound was sewn up and the mouse allowed to remain at room temperature. The TD50 of the leukaemia cells released from the excised fragment was then determined. A second liver sample was removed from the mouse 47 minutes after death and the TD50 determined for the cells in this fragment. The TD50 values obtained were 10 cells and 3·2 cells respectively. It is concluded that the viability loss detected among the cells irradiated in dead mice was due to radiation-induced damage alone and was not contributed to by environmental influences associated with temporary residence of the cells in the tissues of a dead mouse.

TABLE II.—*Log Survival Ratios Among Leukaemia Cells Irradiated in the Livers of Leukaemic Mice soon after Death*

| Dose of radiation (r ; $^{60}$Co gamma rays) | Log survival ratio |
|---|---|
| 800 | $\bar{1}$·44 |
| 1400 | $\bar{2}$·83 |
| 2000 | $\bar{3}$·98 |
| 3000 | $\bar{4}$·79 |

The log survival ratios obtained for leukaemia cells irradiated in dead mice are recorded in Table II, and it is seen from the upper curve of Fig. 1 that there is, again, a linear relationship between log survival ratio and the dose of radiation. From the linear part of the curve, which extrapolates to cut the zero dose axis at about +0·3, the $D_0$ value is approximately 380 r, compared with 165 r for the leukaemia cells irradiated *in vivo* in mice breathing air or oxygen. Thus, for equal survival ratios, the dose required when the cells are under what are assumed to be anoxic conditions is greater than that required when the cells are irradiated in what is assumed to be a moderately well-oxygenated environment, by a factor 2·3 approximately.

*Theoretical radiation survival curves for leukaemia cell populations consisting of known proportions of anoxic and well oxygenated cells*

The linearity of the log survival curves for both anoxic and well-oxygenated leukaemia cells suggests that in each case the cells of the exposed population were remarkably uniform in respect of their environmental oxygen tension. For the cells in dead mice such uniformity is to be expected, since it is inconceivable that foci containing available oxygen could persist among rapidly metabolising cells within an organ whose circulation has ceased. For the cells in mice breathing oxygen or air the apparent uniformity is more surprising : we should expect a proportion of the cells to lie in situations where thrombosis or other vascular accident has given rise to virtually anoxic foci. Areas resembling infarcts, in which both the liver cells and the infiltrating leukaemia cells have undergone necrosis, are indeed to be seen occasionally in advanced leukaemic livers. In the case of many solid tumours, which show extensive areas of necrosis in histological section, it cannot be doubted that many of the malignant cells at the boundary of necrotic zones would be under anoxic conditions. Since it appears probable that the cells of many tumours are heterogeneous in respect of the oxygen tension in their environment it is useful to consider the character of theoretical log survival curves for cell populations consisting mostly of well-oxygenated cells but containing a known proportion of anoxic cells, each variety of cell having a radiosensitivity defined by the appropriate $D_0$ value as determined here for well-oxygenated and anoxic leukaemia cells. It is reasonable to assume that the respective radiosensitivities would not be influenced by the fact that the cells belonged to a mixed population, so that the survival ratio for the total population after any dose of radiation can be expressed as follows :

$$\frac{\text{Surviving well-oxygenated cells} + \text{surviving anoxic cells}}{\text{total initial cell population}}$$

With increasing doses of radiation, the viable anoxic cells, being eliminated at a slower rate than the well-oxygenated cells, will form a rapidly increasing

FIG. 2.—Theoretical radiation survival curves for leukaemia cell populations consisting of mixtures of well-oxygenated and anoxic cells.

proportion of the total surviving population of viable cells. The log survival curve for the total population will thus gradually assume the slope for a pure population of anoxic cells. For example, after exposure to 2500 r of an initial mixed population of $10^6$ cells, consisting of 10 per cent anoxic cells and 90 per cent well-oxygenated cells, there is less than a 50 per cent chance of one viable well-oxygenated cell surviving, whereas about 250 viable anoxic cells would still remain. In Fig. 2 the original separate linear log survival curves for pure populations of well-oxygenated and anoxic leukaemia cells, respectively, are shown. Between these, are shown theoretical curves for mixed populations containing various stated proportions of the two types of cell. It will be seen that populations containing only a small proportion of anoxic cells give a log survival curve slope which does not depart significantly from that for well-oxygenated cells until higher doses of radiation are attained, when the slope changes gradually to that for anoxic cells.

### DISCUSSION

A positive correlation between radiosensitivity and environmental oxygen tension has been demonstrated for a wide variety of cells, including several mammalian tumours (Gray, 1957) ; the observed ratio of the radiosensitivities of anoxic and moderately well-oxygenated normal tissue cells has been similar to that recorded here for leukaemia cells. For example, Howard-Flanders and Wright (1957), using a quite different indicator—the inhibitory effect on bone growth in the mouse tail, found relative radiosensitivity values of 1, 1·97 and 2·56 respectively for the anoxic tail (with occluded blood supply) and the tail in air-breathing and in oxygen-breathing mice. Using visible chromosome damage as an index of radiosensitivity for Ehrlich ascites tumour cells irradiated with X-rays at 18° C. *in vitro*, Deschner and Gray (1959) showed that the relative radiosensitivity of the cells rose rapidly from a minimum value of 1·0 for anoxic cells to about 2·3 for cells in fluid equilibrated with oxygen at a pressure of 20 mm. Hg. With oxygen tensions above this level, radiosensitivity increased more gradually, a value of 3·0 not being attained until the oxygen pressure reached about 400 mm. There is no reason to believe that a similar relationship between oxygen tension and radiosensitivity does not obtain for mammalian tumour cells irradiated *in vivo*, although the environmental oxygen tension of tumour cells *in vivo* would not be expected to be uniform and would not be measurable with the precision possible with an *in vitro* system. Nevertheless, the results with mouse ascites cells (Deschner and Gray, 1959) and other results with bacteria (Alper and Howard-Flanders, 1956) make it probable that the range of oxygen tensions over which we should expect major alteration of the radiosensitivity of mouse leukaemia cells *in vivo* is from zero to about 20 mm. Hg. The range with which we are concerned thus lies distinctly below the tension (40 mm.) normally found in the veins of an air-breathing mammal.

The local tissue oxygen tension for any small group of tumour cells *in vivo* cannot at present be ascertained by direct measurement, although it is possible to calculate theoretical values from various assumptions and data. Such values have been calculated (Thomlinson and Gray, 1955) for foci within squamous carcinomas of human lung, and have been strikingly correlated with the actual spatial relationships of necrotic foci and capillaries as seen in histological sections of these tumours. The complexity of the factors influencing the oxygen tension in the vicinity of tumour cells *in vivo*, and the effects of raising the partial pressure of oxygen respired have been discussed in great detail by Churchill-Davidson, Sanger and Thomlinson (1957). These considerations cannot, however, provide a reliable assessment of the proportion of viable tumour cells which are actually anoxic in man or animals breathing oxygen at normal or supranormal pressures. The theoretical curves shown in Fig. 2 suggest that the proportion of mouse leukaemia cells in the livers of leukaemic mice breathing air or oxygen which are anoxic, is certainly less than 1 per cent and possibly no greater than 0·1 per cent. The proportion of anoxic cells in other tumours, those showing more widespread vascular disturbance, may very well prove to be greater.

It is clear that the relative radioresistance of anoxic tumour cells is such that the presence of these cells in a tumour *in vivo* would be expected substantially to diminish the effectiveness of tumour radiotherapy. It is, therefore, important to discuss certain theoretical considerations concerning the possible incidence of anoxic tumour cells *in vivo*. It is certain that vascular occlusion frequently leads to death of cells, often involving quite large volumes of tumour tissue. Such large-volume necrosis supposedly results from total deprivation of the metabolic requirements of the cells, including glucose, amino acids, vitamins and other growth requirements, as well as oxygen ; the accumulation of waste products also may contribute to the necrosis. The predicament of such grossly deprived cells is sooner or later lethal, and their temporary survival in a tumour would have no influence on its radiocurability. The cells whose relative radioresistance would be of importance to radiocurability are those which are almost or actually anoxic but which nevertheless have their viability preserved over the period of time required for them to reproduce over several generations. This situation implies a differential interference with cell requirements, such that adequate amounts of glucose and other growth factors continue to be supplied, while available oxygen falls very severely. We do not know whether the tissue fluid commonly attains a composition which permits these conditions to prevail, and it is clear that more information is required before it can be assumed that groups of tumour cells may pass through a fairly prolonged period of severe hypoxia *in vivo* and later assert themselves as the progenitors of a massive tumour cell population. Our results suggest that such anoxic cells are uncommon, even in the heavily infiltrated leukaemic mouse liver, where anoxic conditions might be expected.

Although our results suggest that anoxic cells are unlikely to form more than a small proportion of the total malignant cell population in an air- or oxygen-breathing mouse, it should be appreciated that even a very small proportion of such cells could very significantly affect the dose of radiation required to eliminate the growth potential of a large population of tumour cells. A tumour 2 cm. in diameter and consisting half of stroma and half of tumour cells (mean diameter 12·6 $\mu$), with only half the tumour cells capable of reproduction, would contain about $10^9$ reproductively intact malignant cells. Now if each of these cells is capable of regenerating a fresh tumour, a survival ratio of about $10^{-10}$ is required for a 90 per cent chance of eliminating the total malignant cell population. In Fig. 3, the linear survival curves obtained for leukaemia cells irradiated under well-oxygenated and anoxic conditions, respectively, have been extrapolated to very low survival rates. It will be seen that a survival ratio of $10^{-10}$ would be expected after exposure of the malignant cell population to a $^{60}Co$ gamma radiation dose of 4000 r, provided that all the cells were under well-oxygenated conditions at the time of irradiation. If, however, $10^5$ (0·01 per cent) of the cells were under anoxic conditions during exposure, the curve for anoxic cells indicates that after exposure to 4000 r there would be about a 90 per cent chance of one or more reproductively intact cells surviving the irradiation. $10^5$ cells, of the size given, occupy a volume of only 0·1 c.mm. This very small volume of anoxic cells, present in a tumour of 2 cm. diameter, would thus seriously militate against eradication of the tumour by this dose of radiation, and be responsible for reducing an expected 90 per cent cure rate to about 10 per cent. If all the tumour cells were anoxic during irradiation, as would be the case if the vascular supply of the tumour were to be totally interrupted by pressure or torsion, then about 9000 r would have to be delivered to the tumour before a useful cure rate could be expected.

The $D_0$ value obtained by Puck and Marcus (1956) for human HeLa cells irradiated *in vitro* under well-oxygenated conditions with 230 kV X-rays, was 96 r. Puck, Morkovin, Marcus and Cieciura (1957) found a similar value for

FIG. 3.—Extrapolated survival curves for leukaemia cells irradiated under well-oxygenated conditions (in air-breathing mice), and under anoxic conditions (in dead mice).

numerous other human epithelial cell types under similar conditions. Recently Morkovin, and Feldman (1959) pointed out that an error in the original dosimetry requires the value of 96 r to be increased by a factor 1·45 to give an adjusted $D_0$ value of 139 rads. When further increased by the factor 1·25 to allow for the greater R.B.E. of 230 kV X-rays compared with $^{60}$Co gamma rays, the $D_0$ value for human epithelial cells irradiated under well-oxygenated conditions becomes 174 rads of $^{60}$Co gamma radiation, which is not significantly different from the $D_0$ value (161 rads) obtained here for murine leukaemia cells irradiated *in vivo* in air-breathing mice. This remarkably good correlation between the radiosensitivities of human and murine cells suggests that parameters obtained from radio-biological studies of mouse tumours may be directly applicable within the sphere of clinical radiotherapy of tumours. It may be added that consideration of the implications of such parameters should properly precede the use of such procedures as the treatment of human leukaemia by whole-body radiation.

### SUMMARY

A transplantation bio-assay method was used to determine survival ratios among the leukaemia cells released from the livers of leukaemic mice immediately after their exposure to 800, 1400 and 2000 r total-body $^{60}Co$ gamma radiation, a surviving cell being defined as one capable of securing successful transplantation of the leukaemia. No significant difference was demonstrated between the survival ratios obtained for cells from mice breathing 95 per cent oxygen and from mice breathing 95 per cent air during irradiation. None of the survival ratios departed significantly from the linear log survival ratio-radiation dose curve obtained previously for leukaemia cells irradiated in mice breathing air (Hewitt and Wilson, 1959). A linear relationship was demonstrated between log survival ratio and radiation dose for the leukaemia cells irradiated under anoxic conditions (in recently killed mice). Comparison of the log survival curves for cells irradiated in mice breathing air or oxygen and for the anoxic cells showed that the latter were more radioresistant by a factor 2·3. The slope of the log survival curve for cells irradiated in living mice was closely similar to that obtained by Puck and Marcus (1956) for human cancer cells (HeLa) irradiated under well-oxygenated conditions *in vitro*.

We are indebted to Miss J. Howarth, B.Sc. (B.E.C.C. Research Physicist) for carrying out all the irradiations done using the " Theratron ", and to Mrs. D. Levy and Miss E. Blake for skilled technical assistance with the biological work. We are grateful to the British Empire Cancer Campaign for a whole-time grant to one of us (H. B. H.) and for financial support to the laboratory of the other (C. W. W.).

### REFERENCES

ALPER, T. AND HOWARD-FLANDERS, P.—(1956) *Nature*, **178**, 978.
CHURCHILL-DAVIDSON, I., SANGER, C. AND THOMLINSON, R. H.—(1957) *Brit. J. Radiol.*, **30**, 406.
DESCHNER, E. E. AND GRAY, L. H.—(1959) *Radiation Res.*, **11**, 115.
GRAY, L. H.—(1957) *Brit. J. Radiol.*, **30**, 403.
HEWITT, H. B.—(1958) *Brit. J. Cancer*, **12**, 378.
*Idem* AND WILSON, C. W.—(1959) *Ibid.*, **13**, 69.
HOWARD-FLANDERS, P. AND WRIGHT, E. A.—(1957) *Brit. J. Radiol.*, **30**, 593.
MORKOVIN, D. AND FELDMAN, A.—(1959) *Ibid.*, **32**, 282.
PUCK, T. T. AND MARCUS, P. I.—(1956) *J. exp. Med.*, **103**, 653.
*Idem*, MORKOVIN, D., MARCUS, P. I. AND CIECIURA, S. J.—(1957) *Ibid.*, **106**, 485.
REED, L. J. AND MUENCH, H.—(1938) *Amer. J. Hyg.*, **27**, 493.
THOMLINSON, R. H. AND GRAY, L. H.—(1955) *Brit. J. Cancer*, **9**, 539.


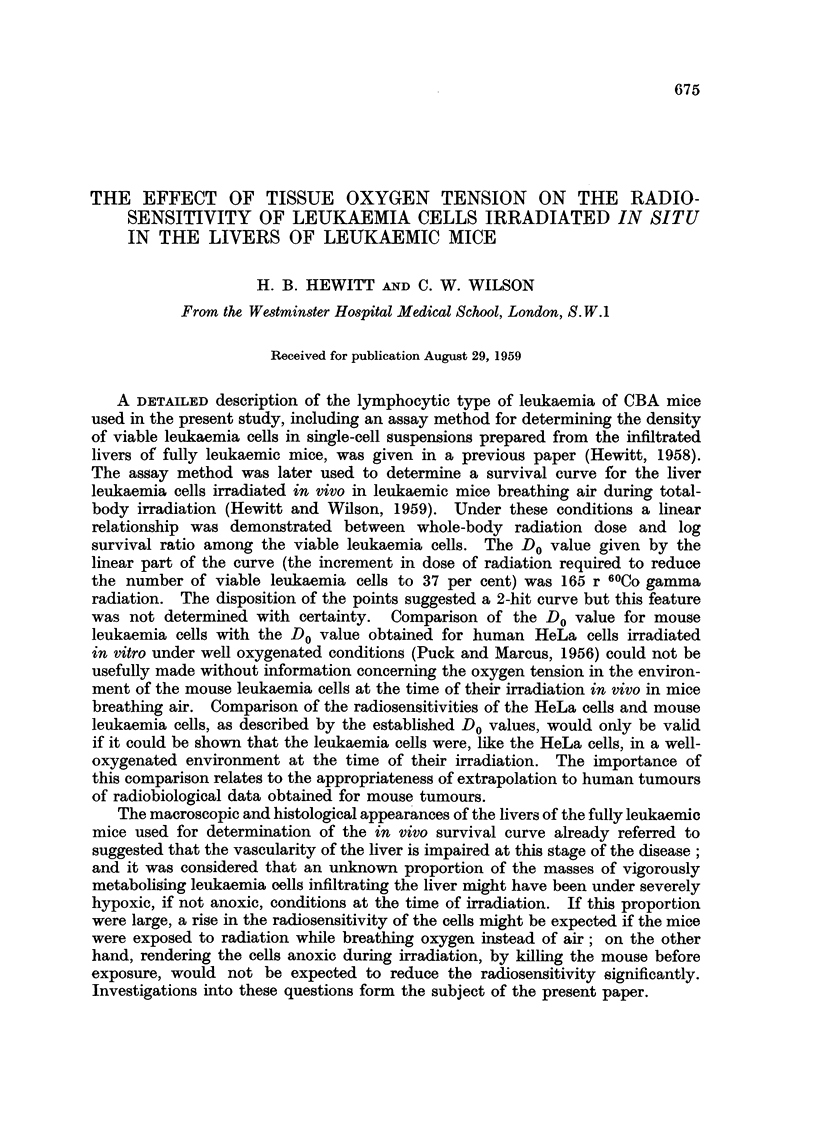

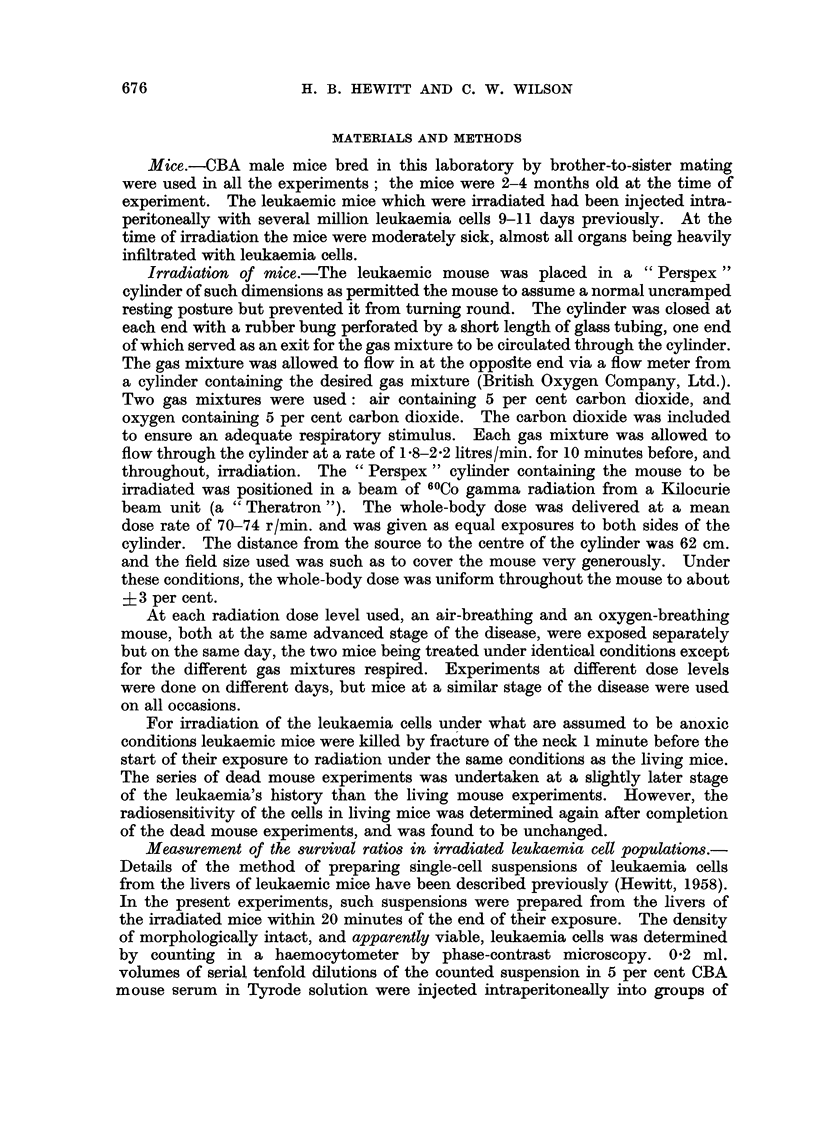

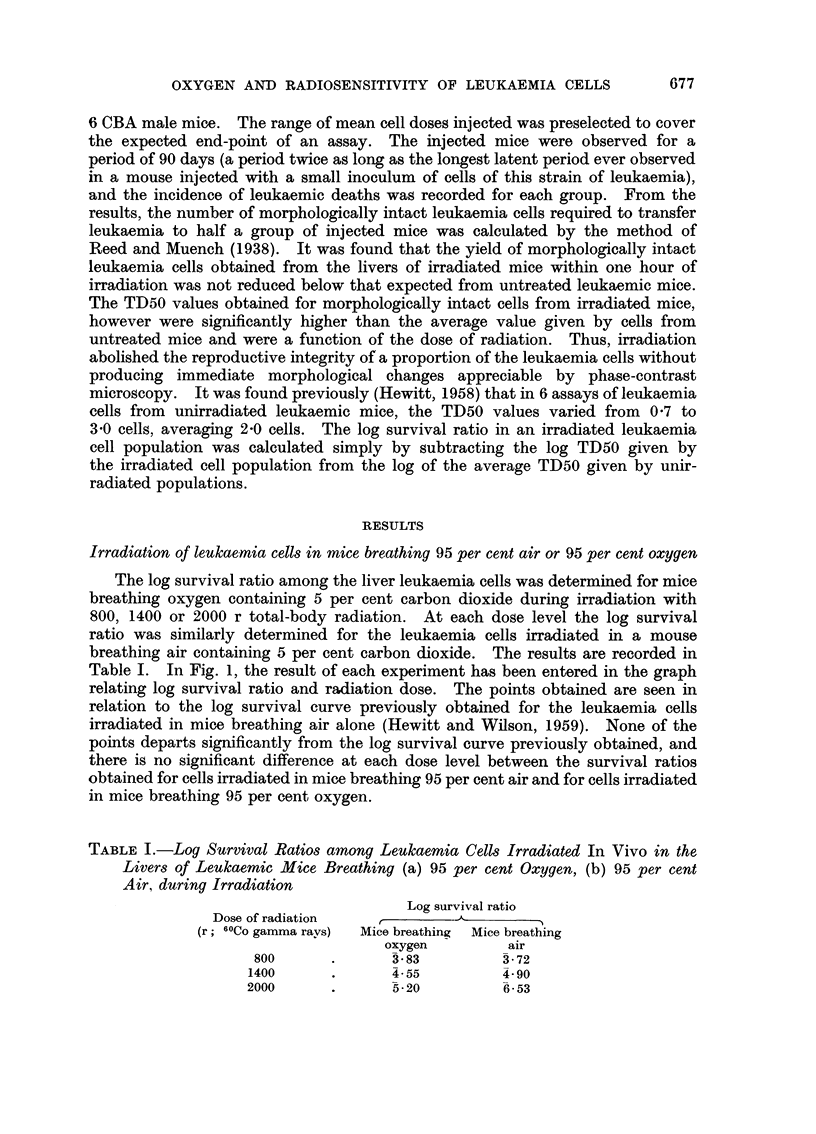

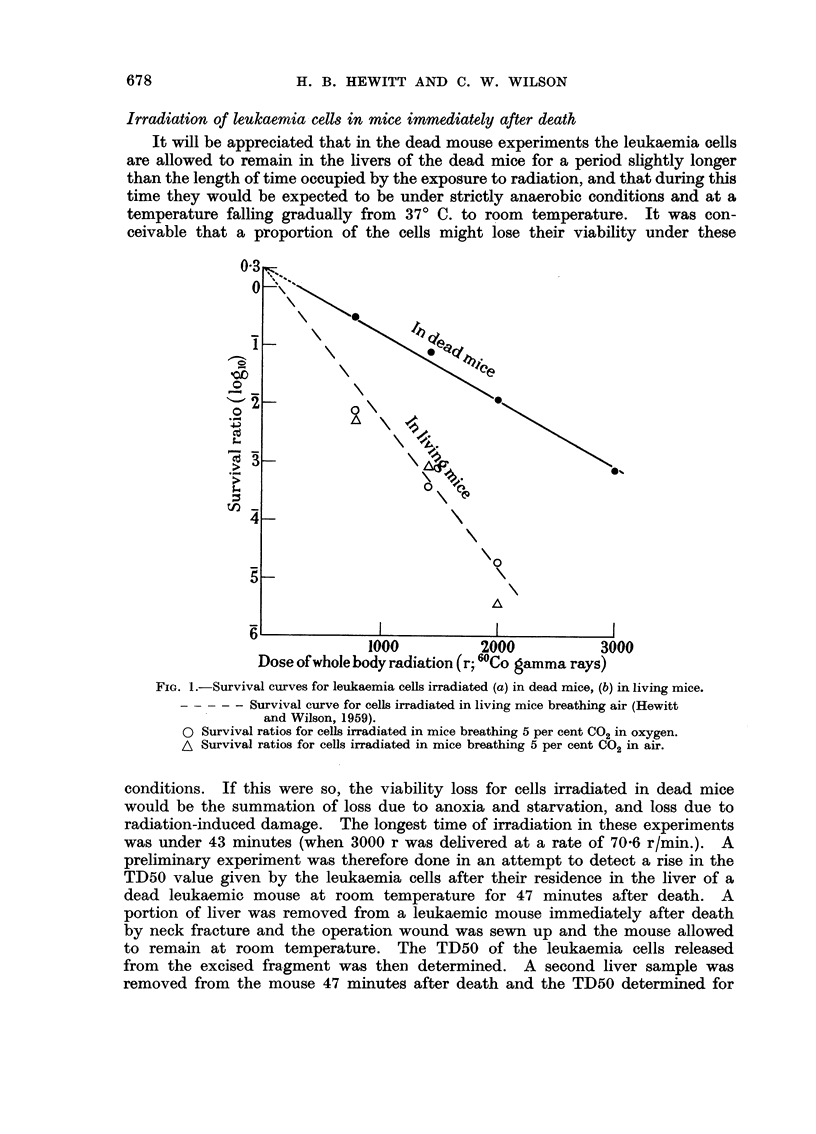

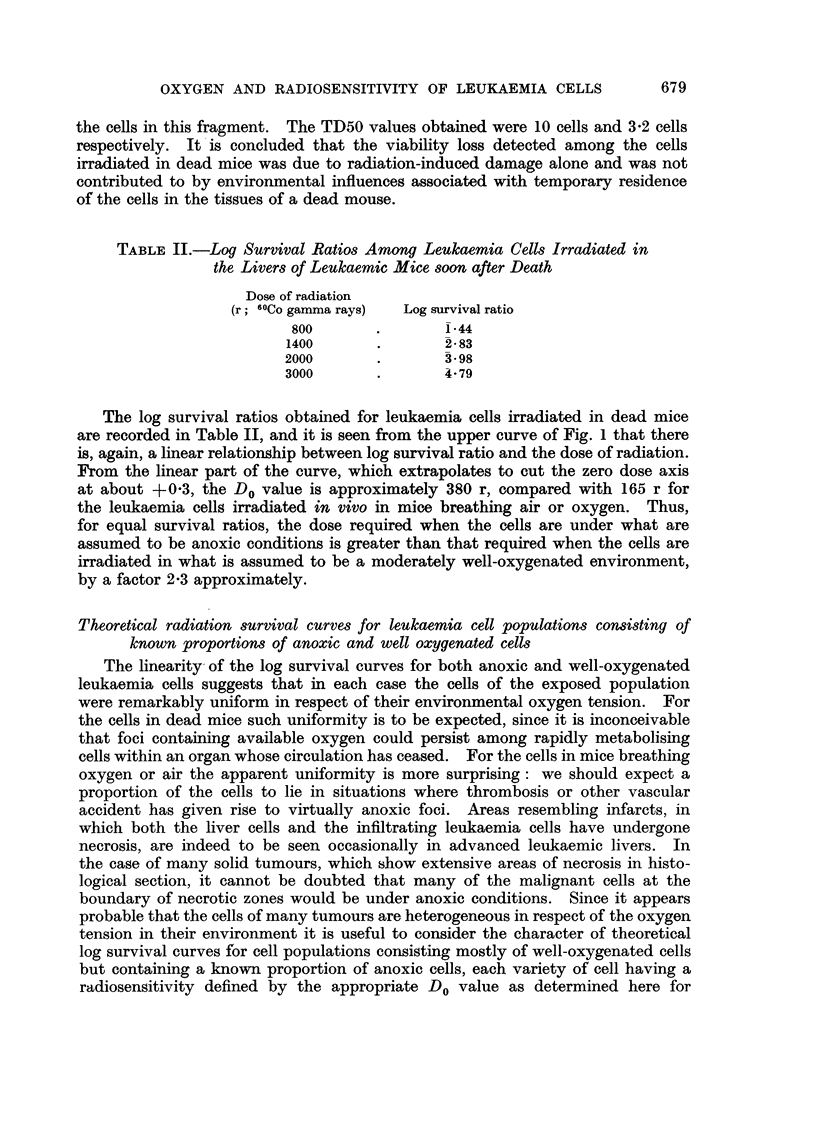

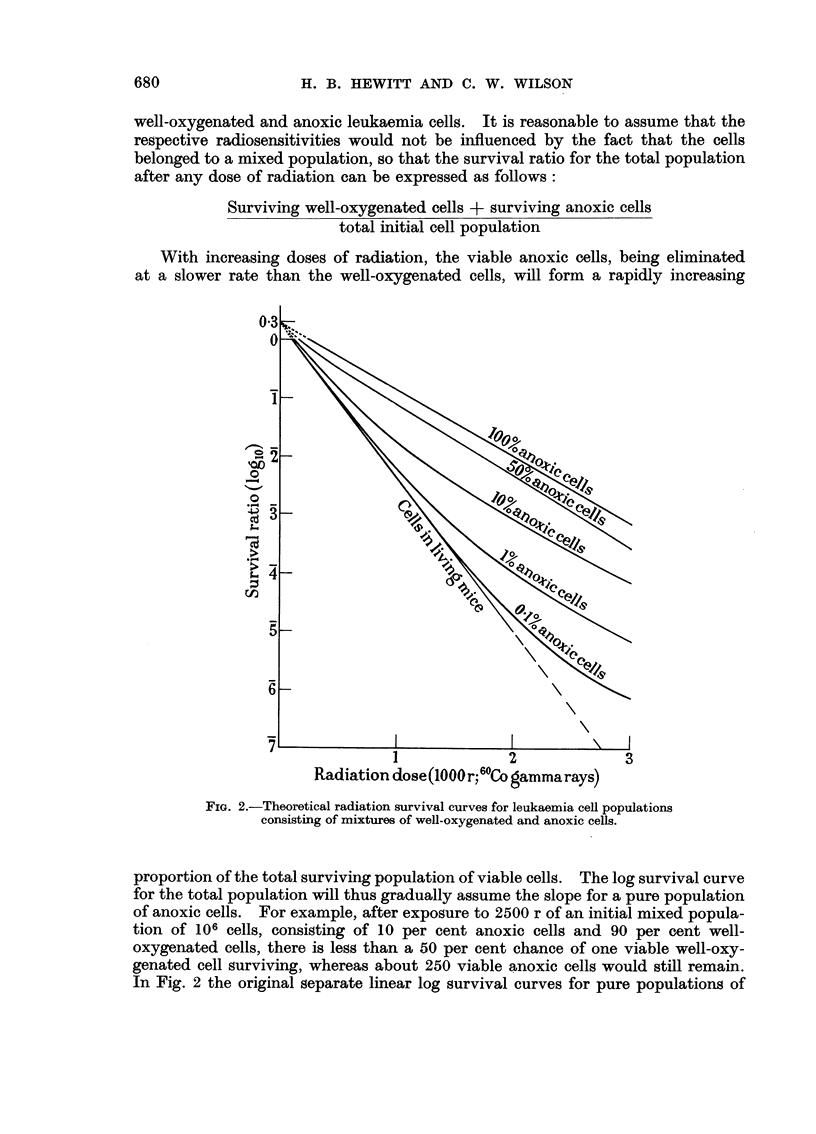

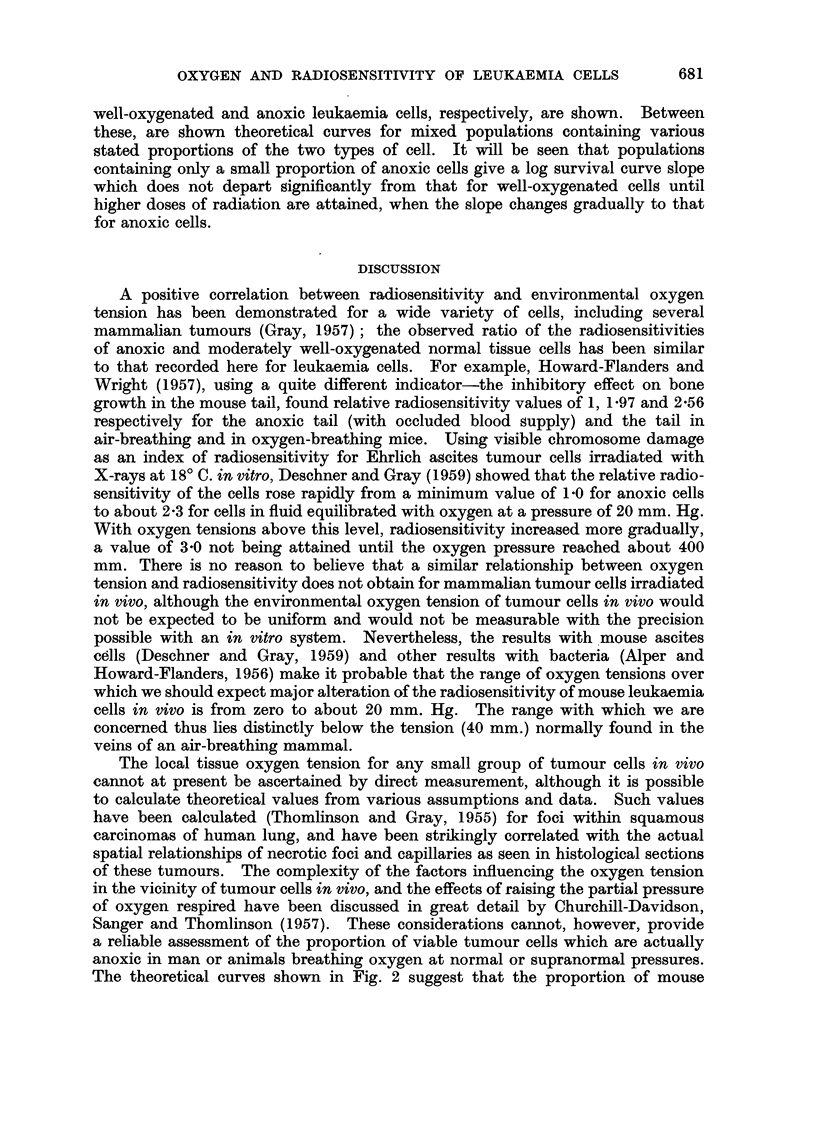

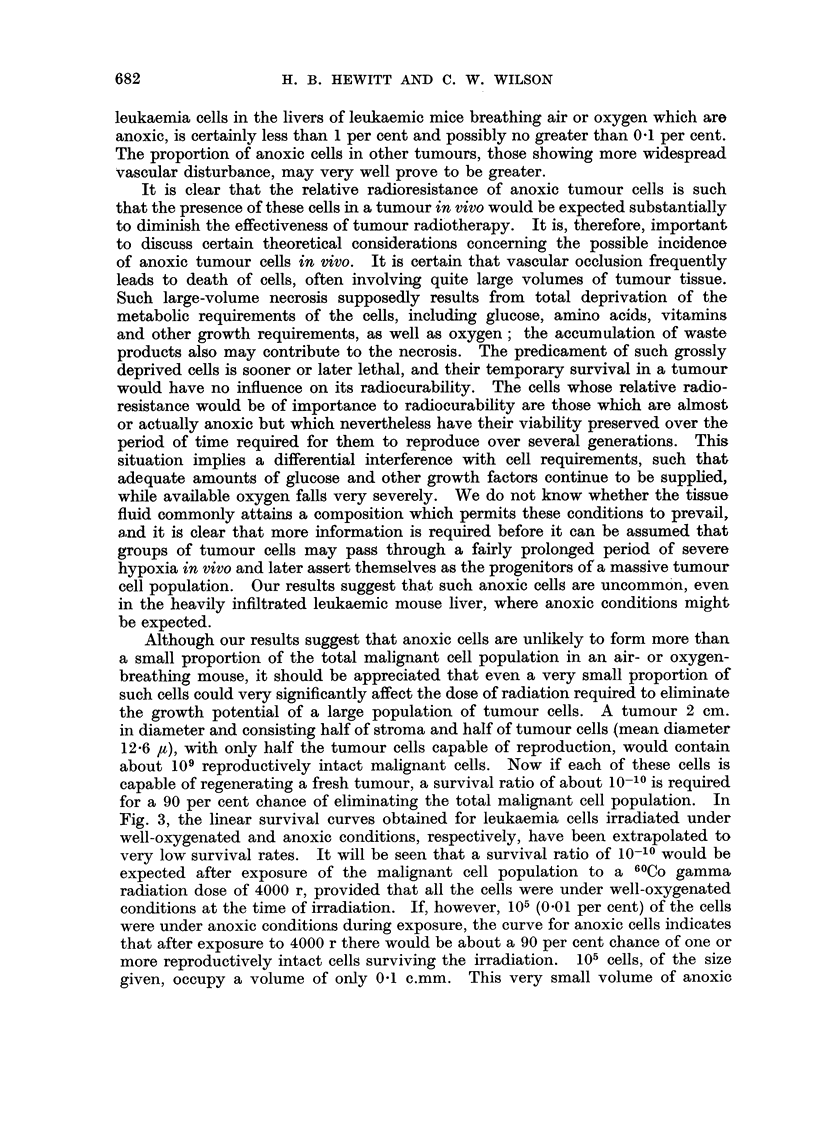

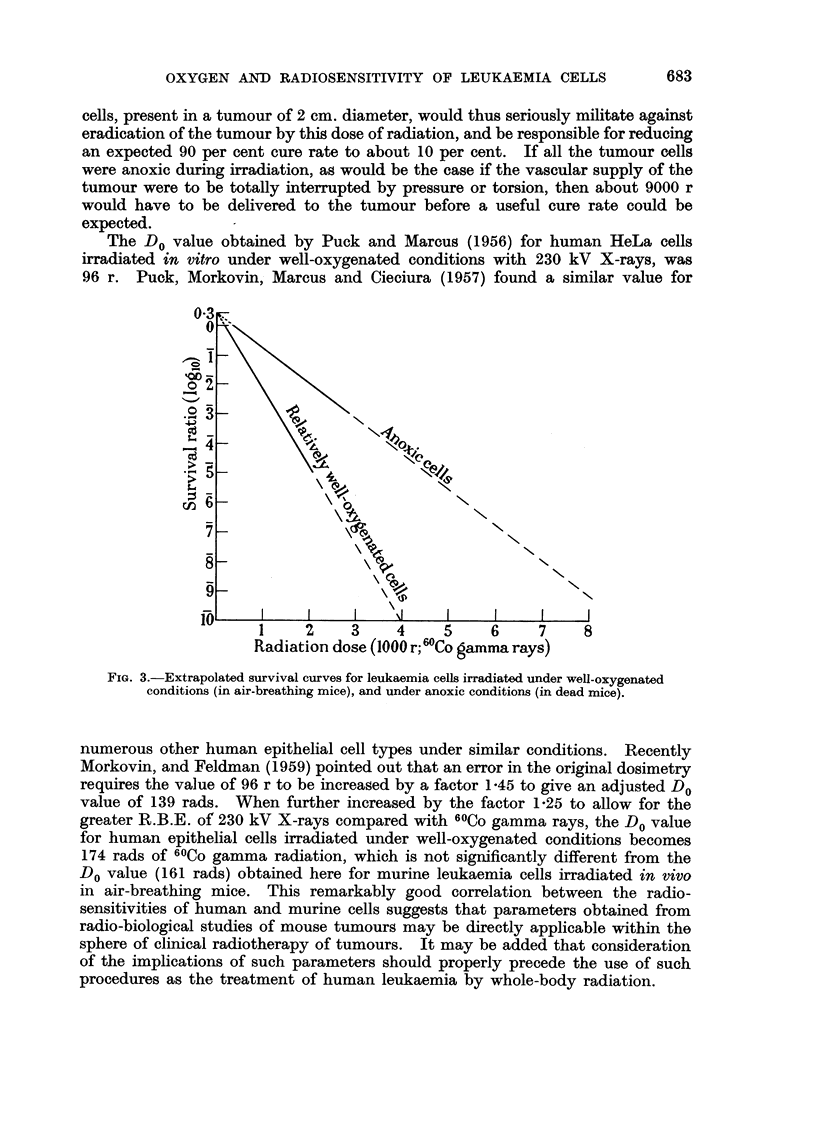

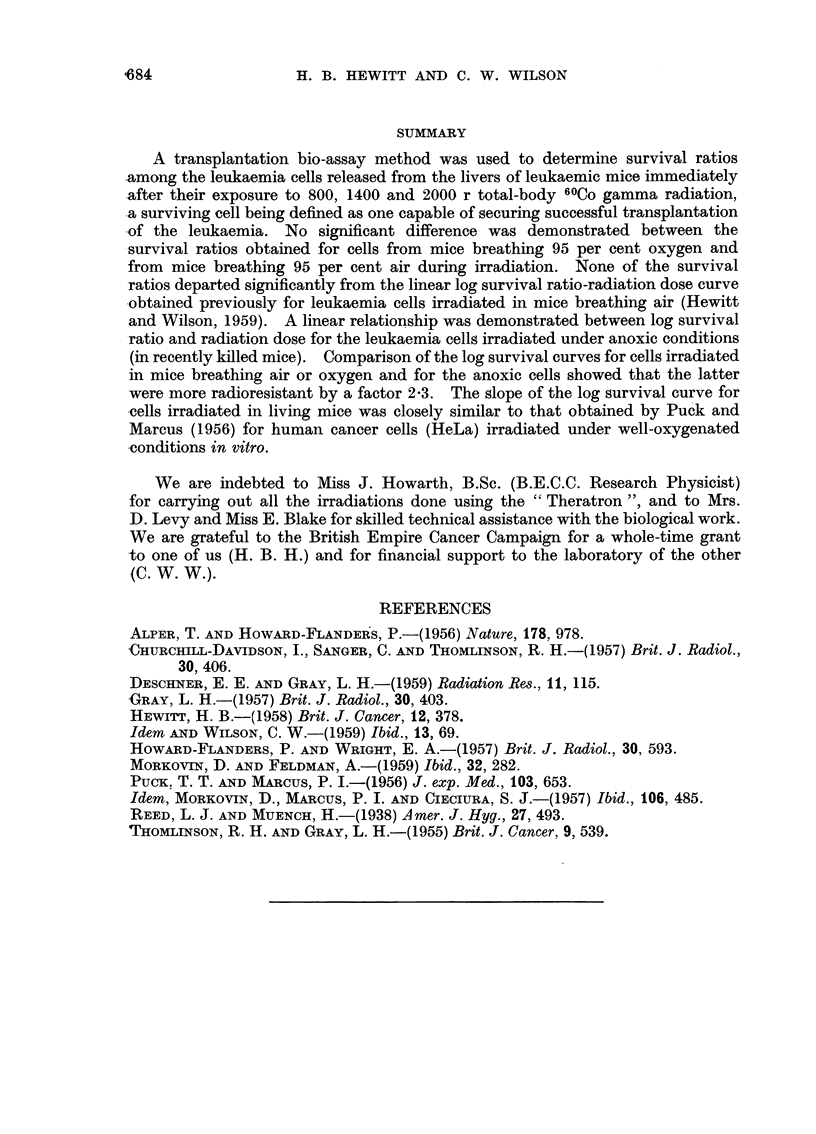


## References

[OCR_00505] CHURCHILL-DAVIDSON I., SANGER C., THOMLINSON R. H. (1957). Oxygenation in radiotherapy. II. Clinical application.. Br J Radiol.

[OCR_00507] DESCHNER E. E., GRAY L. H. (1959). Influence of oxygen tension on x-ray-induced chromosomal damage in Ehrlich ascites tumor cells irradiated in vitro and in vivo.. Radiat Res.

[OCR_00508] GRAY L. H. (1957). Oxygenation in radiotherapy. I. Radiobiological considerations.. Br J Radiol.

[OCR_00510] HEWITT H. B. (1958). Studies of the dissemination and quantitative transplantation of a lymphocytic leukaemia of CBA mice.. Br J Cancer.

[OCR_00513] HOWARD-FLANDERS P., WRIGHT E. A. (1957). The effect of oxygen on the radiosensitivity of growing bone in the tail of the mouse.. Br J Radiol.

[OCR_00516] PUCK T. T., MARCUS P. I. (1956). Action of x-rays on mammalian cells.. J Exp Med.

[OCR_00518] PUCK T. T., MORKOVIN D., MARCUS P. I., CIECIURA S. J. (1957). Action of x-rays on mammalian cells. II. Survival curves of cells from normal human tissues.. J Exp Med.

[OCR_00521] THOMLINSON R. H., GRAY L. H. (1955). The histological structure of some human lung cancers and the possible implications for radiotherapy.. Br J Cancer.

